# Assessment of Cardiorespiratory Interactions during Apneic Events in Sleep via Fuzzy Kernel Measures of Information Dynamics

**DOI:** 10.3390/e23060698

**Published:** 2021-05-31

**Authors:** Ivan Lazic, Riccardo Pernice, Tatjana Loncar-Turukalo, Gorana Mijatovic, Luca Faes

**Affiliations:** 1 Department of Power, Electronic and Communication Engineering, Faculty of Technical Sciences, University of Novi Sad, 21000 Novi Sad, Serbia; gorana86@uns.ac.rs; 2 Department of Engineering, University of Palermo, 90128 Palermo, Italy; riccardo.pernice@unipa.it (R.P.); luca.faes@unipa.it (L.F.)

**Keywords:** cardiorespiratory interactions, information dynamics, apnea, respiratory effort-related arousal (RERA), entropy

## Abstract

Apnea and other breathing-related disorders have been linked to the development of hypertension or impairments of the cardiovascular, cognitive or metabolic systems. The combined assessment of multiple physiological signals acquired during sleep is of fundamental importance for providing additional insights about breathing disorder events and the associated impairments. In this work, we apply information-theoretic measures to describe the joint dynamics of cardiorespiratory physiological processes in a large group of patients reporting repeated episodes of hypopneas, apneas (central, obstructive, mixed) and respiratory effort related arousals (RERAs). We analyze the heart period as the target process and the airflow amplitude as the driver, computing the predictive information, the information storage, the information transfer, the internal information and the cross information, using a fuzzy kernel entropy estimator. The analyses were performed comparing the information measures among segments during, immediately before and after the respiratory event and with control segments. Results highlight a general tendency to decrease of predictive information and information storage of heart period, as well as of cross information and information transfer from respiration to heart period, during the breathing disordered events. The information-theoretic measures also vary according to the breathing disorder, and significant changes of information transfer can be detected during RERAs, suggesting that the latter could represent a risk factor for developing cardiovascular diseases. These findings reflect the impact of different sleep breathing disorders on respiratory sinus arrhythmia, suggesting overall higher complexity of the cardiac dynamics and weaker cardiorespiratory interactions which may have physiological and clinical relevance.

## 1. Introduction

Sleep is a physiological state characterized by noteworthy physiological changes in respiratory and cardiovascular activities, in almost all the stages. As the autonomic nervous system (ANS) activity is strongly related to sleep, several cardiovascular and neurologic disorders may cause sleep disturbances, and conversely disturbances of sleep can produce unbalanced sympathetic or parasympathetic modulation of the cardiovascular functions [1,2]. Sleep-related breathing disorders represent an increasingly frequent problem affecting sleep which lead to alterations in the neural and cardiovascular effects of sleep. Obstructive sleep apnea (OSA), as the most common type of sleep apnea, is characterized by repetitive total (or partial) collapse of the pharyngeal airway during sleep, with complete cessation of airflow occurring irrespective of continued ventilatory effort [3,4]. Hypopnea is associated with reduced airflow and respiratory effort, and may be accompanied by a decreased oxygen saturation, or a reduction of more than 50% of the airflow lasting at least 10 s [5]. The consequent surges in oxygen desaturation, hypercapnia, and catecholamine have now been implicated in development of hypertension and impairments of the cardiovascular, cognitive or metabolic systems, and potentially linked to myocardial infarction, congestive heart failure, stroke, and diabetes mellitus, with a 4- to 6-fold increased risk of mortality [4,6,7,8,9]. OSA has nowadays an increased incidence within the general population, affecting about 10% to 20% of middle to older aged adults [4,10], and it has been also associated to sleepiness which in turn causes tiredness all-over the day with increased risk of road traffic accidents [6,11]. Another subtle sleep breathing disorder is the respiratory effort–related arousal (RERA), which is characterized by obstructive upper airway airflow reduction not meeting the criteria of apnea or hypopnea, associated with increased respiratory effort that resolves with the appearance of arousals [12].

The identification of presence and severity of sleep disorders requires comprehensive sleep evaluation through overnight recordings of multiple physiological signals in a controlled environment. Monitoring and measuring respiration during sleep has undergone many advances in the last decades in terms of quality and validity. Polysomnography allows the synchronous acquisition of multiple physiologic parameters during sleep, e.g., electroencephalogram, electro-oculogram, electromyogram, electrocardiogram (ECG), and pulse oximetry, as well as airflow and respiratory effort, to permit a comprehensive evaluation of the underlying causes of sleep disturbances [13]. Despite the improvement in diagnostic standards and recognition of sleep-disordered breathing, several limitations still need to be overcome [14]. In particular, the research in this field is now focusing on novel analyses techniques capable of a combined assessment of the multiple physiological signals acquired during sleep, since studying both the individual dynamics and the interactions between multiple variables can provide additional insights into breathing disordered events and the associated impairments [15,16,17,18].

Heart rate variability (HRV) [19] has been extensively analyzed during sleep for an early detection and prevention of cardiovascular events associated with apnea or other breathing-related disorders [15]. Additional insights are provided by the investigation of the mutual influence of the cardiac and respiratory oscillations on their respective onsets, i.e., the cardio-respiratory coordination and phase-coupling, which are strongly affected during sleep apnea events [17,20]. More recently, the research on neural and cardiovascular dynamics during sleep has seen a shift of paradigm from univariate analysis (e.g., HRV from ECG recordings) and cardiorespiratory interactions through simple non-dynamical analyses [17,20] to dynamic approaches able to provide a joint characterization of cardiac, respiratory and neural time series, based on multivariate and non-causal analyses. This paradigm shift exploits the network physiology approach which defines the human body as the interconnection of multiple nodes (i.e., organs) representing the activity of a specific physiological system that exhibits autonomous dynamics with its own regulatory mechanisms, but is at the same time strongly interconnected to other diverse systems [16,18]. A reliable reconstruction of the network structure based on the detection of directional effects and the assessment of joint dynamics of the physiological processes can be achieved by employing the tools of information dynamics [21,22,23,24], already successfully used for analysis of the cardiorespiratory information dynamics and the brain-heart interactions [25,26]. Such an approach aims to describe the joint dynamics of physiological processes, assessing to what extent the contributions of different processes can be quantified and tied to a target process. A dynamical approach takes into account the flow of time by studying how much the past system history contributes to resolve the uncertainty about its present state [24]. The information dynamics framework provides measures quantifying the ‘information content’ of individual variables or collections of variables, and the information exchange between variables [24]. In this sense, the information storage (IS) assesses the information contained in the past states of a target process that can be used to predict its present state. Transfer entropy (TE) quantifies the information transfer, i.e., the information carried by the present of the target that can be predicted by the past of a putative driver process above and beyond the part that was predicted by the past of the target. The internal information (assessed by the conditional self entropy (cSE) measure) provides insights on the internal dynamics of the target process. The cross-entropy (CE) quantifies the cross information, i.e., the part of the information carried by the present of the target that can be predicted by the past of the driver [27]. Finally, the predictive information is a quantity which subsumes the previous ones, quantifying how much the uncertainty about the current state of the target system is reduced by the knowledge of the past states by the overall multivariate system [27].

Although the tools of information dynamics are well-defined from a theoretical point of view, their practical implementation remains challenging due to the need of estimating entropy measures in high-dimensional embedding spaces populated by a limited number of process realizations. This issue emerges even more in the analysis of time series acquired during manifestations of sleep disorders, which are typically characterized by short episodes often associated with non-linear dynamics and non-stationary behaviors. To face these issues, the present work introduces an approach to quantify cardiorespiratory interactions during sleep-related breathing disorders which is based on: (i) the design of a processing pipeline which allows extracting observations of the considered dynamic processes (i.e., heart rate and respiration) from short stationary time windows centered around single episodes of a given breathing disorder, and then forming an overall observation matrix from several episodes of the same disorder; (ii) the implementation of two strategies for decomposing the predictive information of the target process (i.e., HRV) into amounts related to the target dynamics (i.e., IS or cSE measures) and to the respiratory driver dynamics (i.e., TE or CE measures); and (iii) the introduction of a new approach for the estimation of entropy measures which improves kernel-based methods weighing probability estimates by means of a fuzzy membership function. Following a preliminary investigation carried out in a conference paper [28], the analysis is carried out in a large group of patients reporting repeated episodes of arousals due to non-apneic and apneic events including respiratory effort related arousals (RERAs), by computing the information dynamic measures before, during and after the breathing disorder and compared to control segments of undisturbed sleep. The research has been carried out in order to improve our comprehension of cardio-respiratory interplay during different apneic events in sleep, including also arousal events like RERA, without signs of oxygen desaturation. These types of subtle respiratory events are recently being more recognized in clinical evaluation of sleep pathophysiology for their connection with presentation of significant, sleep apnea symptoms and potential development of more serious apneic events later [29,30].

The paper is organized as follows. Section 2 describes the database and the methodology used for data preprocessing, the extraction of time series and measures for assessing cardiorespiratory information dynamics using fuzzy kernel entropy estimator. Section 3 and Section 4 describe the results and their physiological discussion, respectively, while Section 5 summarizes the conclusions.

## 2. Materials and Methods

### 2.1. Database

**Figure 1:**
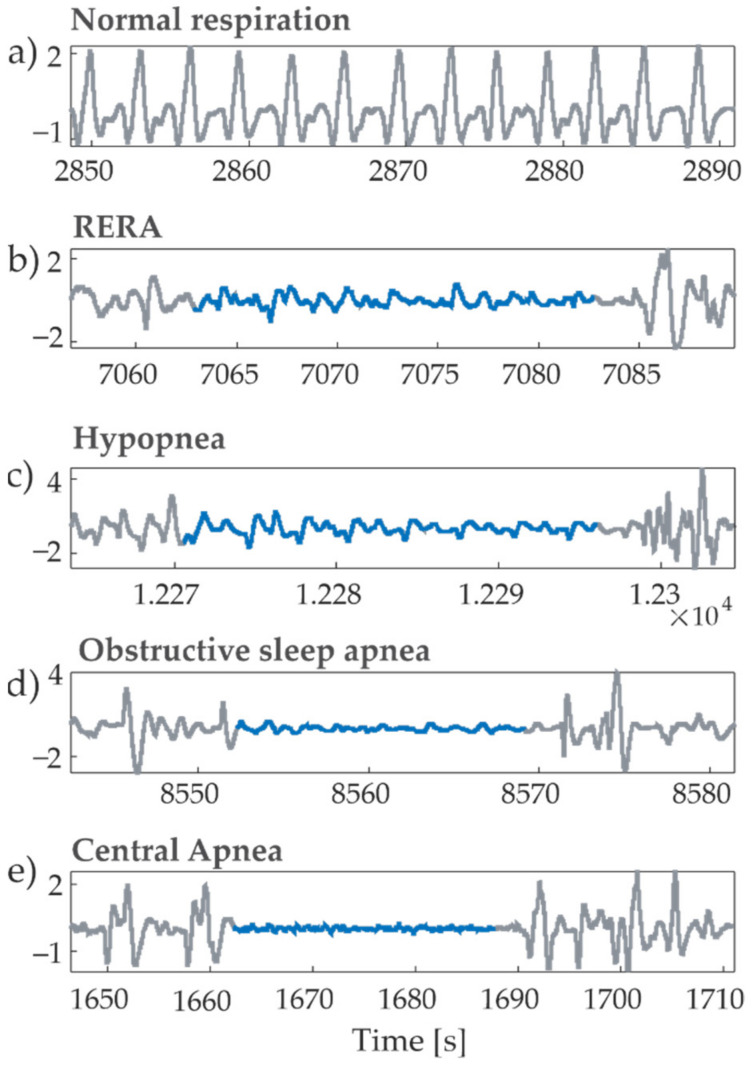
Exemplary windows of the respiratory airflow signal recorded for different breathing effort events during sleep: (**a**) normal respiration; (**b**) RERA; (**c**) hypopnea; (**d**) obstructive sleep apnea; (**e**) central apnea. The signal parts in blue correspond to the labelled events.

The dataset analyzed in this research is an open-source database provided for the Physionet/CinC Challenge 2018 [31], consisting of overnight polysomnographic recordings acquired on 994 subjects. The accompanying sample-wise annotations indicate the onset and offset of different respiratory-related disorders and sleep stages. The recordings are all digitized at 200 Hz and have an average duration of 7.7 (±0.68) hours. As an extension of a preliminary conference paper [28], we focus on the analysis of cardiorespiratory interactions taking into account the airflow respiration signal and the electrocardiogram (ECG) signal. Among the abundance of respiratory events contained in the database, we have focused on respiratory effort-related arousals (RERAs), hypopneas (HAs), obstructive sleep apneas (OAs) and central apneas (CAs). Examples of airflow signals during normal breathing condition and each of the breathing disorders are shown in Figure 1.

### 2.2. Preprocessing and Extraction of Time Series

**Figure 2:**
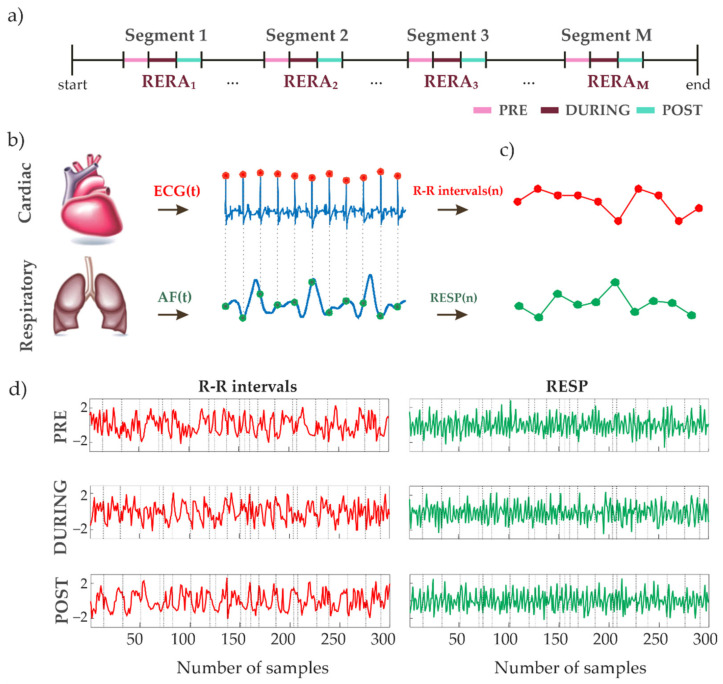
Schematic representation of the methodological processing steps used for forming observation matrices: (**a**) analyzed segments, which included the whole duration of the event (DURING) and 20 s epochs immediately before (PRE) and after (POST) it; (**b**) ECG and airflow signals, respectively acquired from the cardiac and the respiratory system (R-peaks and the corresponding AF value are highlighted in red and green, respectively), from which RR and RESP time series are extracted; (**c**) extraction of an instance of synchronous segments consisting of RR intervals and AF amplitude values; (**d**) repetition of more instances for each given experimental condition (PRE, DURING, POST) to form 300-point time series; vertical lines separate different segments. Note that the segments are joined in (**d**) for visualization purposes only, since the realizations of the RR and RESP patterns used for information analysis are extracted separately from each segment (see Section 2.4).

The methodology followed in this study for extracting from ECG and AF signals the time series to be analyzed with information theory is described in this subsection. The algorithms were developed using MATLAB R2018a (MathWorks, Natick, MA, USA). The analyzed data consist of the signals acquired during nighttime in correspondence with the considered respiratory effort events (i.e., RERAs, HAs, CAs and OSAs). The segments for each respiratory event during sleep were identified according to the provided annotations, with an additional restriction to exclude rapid eye movement (REM) sleep periods, as defined by the accompanying sleep stage annotations. Specifically, for any given type of adverse event we considered a time window including the whole duration of the event as well as the epochs of 20 s immediately before (PRE) and after (POST) the event (see, e.g., Figure 2a). The raw data and annotations were parsed using the WFDB toolbox from PhysioNet [32,33].

The airflow and ECG signals, reflecting the function of two dynamical systems (the respiratory and cardiac, respectively), were monitored and analyzed in the defined periods (Figure 2b). Both signals were filtered with a low pass FIR filter using a Blackman window function in order to obtain the frequency components of interest. The cut-off frequencies of the low-pass filters for the airflow signal and for the ECG signal were set to 6 Hz and 40 Hz, respectively. In order to reduce the issues of sharp changes in the airflow amplitude range, the signal was energy-normalized in 1-min-long windows prior to filtering. Finally, both signals were standardized through a z-score like transformation using the median and interquartile range values.

In order to obtain the RR interval values from the ECG, two R peak detection algorithms [34,35] were used and their results compared after an adaptive filtering procedure for removal of ectopic beats [36]. The performance of the detectors was evaluated based on comparison of detected R peaks and their positions before and after adaptive filtering. The algorithm requiring less corrections in the adaptive filtering procedure was selected [37]. Comparing the RR intervals time series (herein referred as RR) before and after the adaptive filtering allowed the detection and removal of corrupted signal segments. The respiration time series (RESP) were obtained by sampling the airflow signal at the times of R peak occurrence denoting the onset of each detected RR interval (Figure 2b).

The described measurement procedure allowed to extract multiple instances of synchronous segments consisting of RR intervals and AF amplitude values. The segments were extracted for each subject and respiratory (arousal) event as schematically represented in Figure 2c for one of these instances. Each of these synchronous segments were further processed through an autoregressive high-pass filter to ensure fulfillment of the stationarity criteria [21]. The RR and RESP samples taken from these segments (Figure 2d) were then employed to build the observation matrix needed for the estimation of the information dynamics measures; details about the formation of this matrix and the subsequent estimation of the information measures are given in Section 2.4. Additionally, for each included subject CONTROL segments were obtained from undisturbed sleeping periods without any tied respiratory effort events, and distant at least 10 min apart from the nearest event, so as to be reasonably considered not affected by it.

### 2.3. Cardiorespiratory Information Dynamics

The RR and RESP time series extracted as described in the previous section were analyzed using measures of cardiorespiratory information dynamics obtained employing the framework designed in [24]. The analyses were carried out using modified versions of the functions from the ITS toolbox [22,24,38] in order to calculate the measures of information dynamics with a fuzzy kernel function. Specifically, the cardiorespiratory information dynamics were studied considering a bivariate stochastic process formed by the RR and RESP dynamics, considering the RR as the target process Y and RESP as the driver process X [22,23], and decomposing the predictive information about the RR into contributions related to its dynamics and to the dynamics of the RESP process. The predictive information quantifies the information that the present state of the target process Y shares with the past state of the considered bivariate process $\left\{X,Y\right\}$, and is defined as:

(1) $$P_{Y}=I\left(Y_{n};Y_{n}^{-},X_{n}^{-}\right)=H\left(Y_{n}\right)+H\left(Y_{n}^{-},X_{n}^{-}\right)-H\left(Y_{n},X_{n}^{-},Y_{n}^{-}\right),$$

where $Y_{n}$ is the present of RR, $Y_{n}^{-},X_{n}^{-}$ represent the past of RR and RESP respectively, $I\left(\cdot ;\cdot \right)$ is the mutual information and $H\left(\cdot \right)$ is the Shannon entropy.

**Figure 3:**
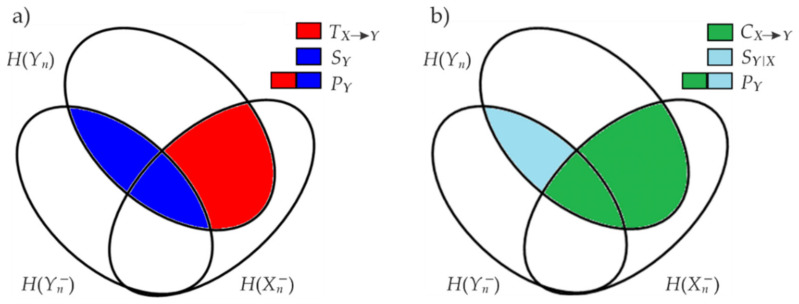
Graphical representation of the information decomposition of the predictive information $P_{Y}$ as (**a**) information storage $S_{Y}$ and information transfer $T_{X\to Y}$ and (**b**) as internal information $S_{Y|X}$ and cross information $C_{X\to Y}$.

The decomposition of the predictive information can be performed in two ways applying the chain rule for mutual information as depicted by the Venn diagrams in Figure 3.

The first decomposition (Figure 3a) computes $P_{Y}$ via the sum of the information stored within the RR ($S_{Y}$) and information transferred into it from RESP ($T_{X\to Y}$) [24] as

(2) $$P_{Y}=S_{Y}+T_{X\to Y},$$

where

(3) $$S_{Y}=I\left(Y_{n};Y_{n}^{-}\right)=H\left(Y_{n}\right)+H\left(Y_{n}^{-}\right)-H\left(Y_{n},Y_{n}^{-}\right)$$

is the self-entropy that quantifies the information carried by the present of the RR that is predicted by its own past [24], and

(4) $$T_{X\to Y}=I\left(Y_{n};X_{n}^{-}|Y_{n}^{-}\right)=H\left(Y_{n},Y_{n}^{-}\right)-H\left(Y_{n}^{-}\right)+H\left(X_{n}^{-},Y_{n}^{-}\right)-H\left(Y_{n},X_{n}^{-},Y_{n}^{-}\right)$$

is the transfer entropy that measures the part of information carried by the present of RR that is predicted by the past of RESP beyond the part that was predicted by the past of RR; I(·;·|·) denotes the conditional mutual information.

The alternative decomposition (Figure 3b) assesses the predictive information via the sum of the cross information between RESP and RR ($C_{X\to Y}$) and the internal information of RR given RESP ($S_{Y|X}$) [24] as:

(5) $$P_{Y}=C_{X\to Y}+S_{Y|X},$$

where

(6) $$C_{X\to Y}=I\left(Y_{n};X_{n}^{-}\right)=H\left(Y_{n}\right)+H\left(X_{n}^{-}\right)-H\left(Y_{n},X_{n}^{-}\right)$$

denotes the CE which quantifies cross-information as the part of information carried by the present of the RR that is predicted by the past of RESP and

(7) $$S_{Y|X}=I\left(Y_{n};Y_{n}^{-}|X_{n}^{-}\right)=H\left(Y_{n},X_{n}^{-}\right)-H\left(X_{n}^{-}\right)+H\left(X_{n}^{-},Y_{n}^{-}\right)-H\left(Y_{n},X_{n}^{-},Y_{n}^{-}\right)$$

is the conditional self-entropy which measures the internal information as the part of information carried by the present of RR that is predicted by the past of RR beyond the part that was predicted by the past of AF. The decomposition (2) is more commonly applied since it evidences the concepts of IS and information transfer which are very well established in the study of information dynamics [39]. On the other hand, the alternative decomposition (5) has been shown to provide a measure of information shared between two processes (i.e., the CE) which is meaningful in the case of prevalent unidirectional interactions, and a useful measure of the strength of the internal dynamics relevant to the analyzed target process (i.e., the conditional self-entropy) [24].

### 2.4. Fuzzy Kernel Entropy Estimation

**Figure 4:**
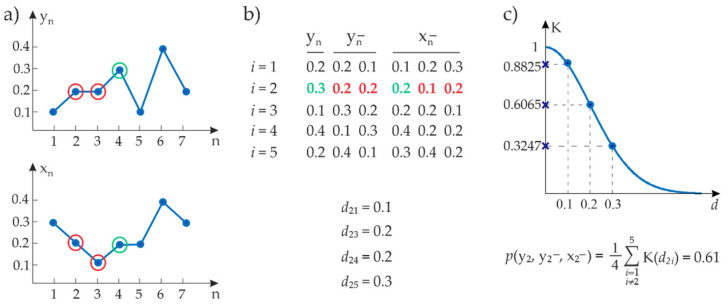
Exemplary representation of the fuzzy probability estimation procedure: (**a**) representative segments of generic time series Y and X of *N* = 7 samples, with the samples forming the second observation of the present value of Y, $Y_{n}$, and of the past values of X and Y, $X_{n}^{-}$ and $Y_{n}^{-}$, encircled in green and red; (**b**) observation matrix formed placing in the *i*th row the samples of the *i*th observation of $Y_{n},X_{n}^{-},X_{n}^{-}$ (Chebyshev distances between the second observation and the other observations are indicated below the matrix); (**c**) Gaussian kernel function and probability estimate for the second observation calculated as the average of the kernel function applied to the Chebyshev distances between the second and all other observations.

The RR and RESP samples taken from the segments as defined in Section 2.2 were employed to form the observation matrix used to estimate the information dynamics measures described in Section 2.3. This matrix was built, for each subject and experimental condition, as reported in the schematic representation of Figure 4. First, the past history of the target and driver processes was approximated using a finite number m of lagged components (in this work, we set m=2 as common in short-term variability analysis [21,40]), i.e., $Y_{n}^{-}≅Y_{n}^{m}=\left[RR_{n-1},RR_{n-2}\right]$, $X_{n}^{-}≅X_{n}^{m}=\left[AF_{n},AF_{n-1},AF_{n-2}\right]$; note that a zero-lag effect was included in $X_{n}^{-}$ to account for fast effects of respiration on the heart period [41]. Using this approximation, each row of the observation matrix contains the realizations of $Y_{n}$, $Y_{n}^{-}$ and $X_{n}^{-}$, in the first column, Columns 2–3, and Columns 4–6, respectively. The rows of the matrix thus gather observations from one of the segments belonging to the analyzed experimental condition (PRE, DURING or POST), and the matrix was formed spanning all data segments corresponding to the several instances of that condition. The overall observation matrix obtained in this way was then split in sub-matrices each containing 300 RR and RESP samples. The length corresponding to 300 heartbeats is a typical choice in short-term cardiovascular variability [40,42,43]; an example is reported in Figure 2d. A subject was disregarded if the required number of points in the observation matrix had not been achieved for at least one of the conditions (PRE, DURING or POST) in the above-described procedure.

The measures of information dynamics were estimated from each observation (sub)matrix using a model-free kernel estimator which extends the kernel approach used to compute the sample entropy [21,44] to the bivariate analysis applied here. The kernel entropy estimator reconstructs the probability distribution of a random variable at each observed point and then derives the related entropy measure using ensemble averages. Specifically, taking into account the rows of the observation matrix (Figure 4b), we compare them using the Chebyshev distance (i.e., maximum norm), where the distance between two vectors is obtained as the maximum of the absolute differences of its components. The kernel estimate of the probability distribution for the *n*th observation $v_{n}$ of a generic vector variable V is computed from N observations of the variable as:

(8) $$p\left(v_{n}\right)=\frac{1}{N-1}\overset{N}{\underset{i=1,i\neq n}{\sum }}K\left(d_{ni}\right)$$

where $d_{ni}=v_{n}-v_{i}$ is the Chebyshev distance between the *n*th and *i*th observations of V, and K is the kernel function (Figure 4c). The comparison excludes self-matching (i≠n). In this work, a Gaussian fuzzy membership function was used as the kernel function in order to allow weighted distancing between the observations [45]:

(9) $$K\left(x;\sigma ,\;c\right)=e^{\frac{-{\left(x-c\right)}^{2}}{2\sigma^{2}}}$$

where σ denotes the standard deviation and c the mean of the Gaussian function. In this study, to cope with normalized time series the parameter σ was fixed to 0.2 whereas c was set to 0.

In our work, the role of the generic variable V is taken by the variables $Y_{n}$ (present RR), $Y_{n}^{-}$ (past RR) and $X_{n}^{-}$ (present and past AF) which appear in Equations (1)–(7), whose realizations $y_{n}$, $y_{n}^{-}$ and $x_{n}^{-}$ form the rows of the observation matrix. These realizations can be used to compute kernel estimates of the measures of information dynamics by using average probabilities in the entropy definition, i.e., $\hat{H}\left(V\right)=-\ln\langle p\left(v_{n}\right)\rangle$, where 〈·〉 denotes average over realizations [21]. With this approach, the predictive information (1), the information storage (3), the information transfer (4), the cross-information (6) and the internal information (7) are estimated as:

(10) $${\hat{P}}_{Y}=\ln\frac{\langle p\left(y_{n},y_{n}^{-},x_{n}^{-}\right)\rangle }{\langle p\left(y_{n}\right)p\left(y_{n}^{-},x_{n}^{-}\right)\rangle }$$

(11) $${\hat{S}}_{Y}=\ln\frac{\langle p\left(y_{n},y_{n}^{-}\right)\rangle }{\langle p\left(y_{n}\right)p\left(y_{n}^{-}\right)\rangle }$$

(12) $${\hat{T}}_{X\to Y}=\ln\frac{\langle p\left(y_{n},y_{n}^{-},x_{n}^{-}\right)p\left(y_{n}^{-}\right)\rangle }{\langle p\left(y_{n},y_{n}^{-}\right)p\left(y_{n}^{-},x_{n}^{-}\right)\rangle },$$

(13) $${\hat{C}}_{X\to Y}=\ln\frac{\langle p\left(y_{n},x_{n}^{-}\right)\rangle }{\langle p\left(y_{n}\right)p\left(x_{n}^{-}\right)\rangle },$$

(14) $${\hat{S}}_{Y|X}=\ln\frac{\langle p\left(y_{n},y_{n}^{-},x_{n}^{-}\right)p\left(x_{n}^{-}\right)\rangle }{\langle p\left(y_{n},x_{n}^{-}\right)p\left(y_{n}^{-},x_{n}^{-}\right)\rangle }.$$

When used to determine the conditional entropy, this estimator is usually referred as fuzzy entropy (FuzzyEn) [46] and represents a method already employed for characterization of time series regularities which improves the sample entropy estimator [44]. Here, the improvement brought by the use of fuzzy kernel functions is extended to the estimation of bivariate measures of information dynamics.

### 2.5. Statistical Analysis

The significance level of the kernel-based estimates of the measures of information dynamics was assessed through a surrogate test performed for each pair of RR and RESP time series obtained partitioning the observation matrix in sub-matrices of 300 rows. Specifically, following the procedure described in [47], surrogate time series were generated to determine the statistical significance of the measure: (a) for information storage, random permutation surrogates were constructed for both the RR and RESP time series by shuffling the order of the time series samples; (b) for the internal information, surrogates were constructed by random permutation of the RR while keeping the RESP time series intact; (c) for both the information transfer and cross information measures, the surrogates were constructed by applying the iterative amplitude-adjusted Fourier transform (IAAFT) algorithm on the RR and RESP time series [48]. Assuming a statistical significance of 95% for a one-sided test, a total of 19 surrogates were formed for each time series [49], the maximum value of each entropy measure computed over the surrogate time series was compared to its value attained for the original sequence, and the entropy measure was deemed as statistically significant if the original value exceeded the maximum surrogate value.

In order to perform a comparative analysis across conditions, the statistical significance of the differences between the distributions of each given information measure computed over the PRE, DURING and POST segments was tested using the nonparametric Kruskal-Wallis test, followed by a post-hoc pairwise nonparametric Wilcoxon signed rank test (two-sided test) with the Bonferroni correction. Moreover, pairwise comparisons between the CONTROL and the time-defined segments were made using the Wilcoxon rank sum test. Statistical significance for all the tests was set at *p* < 0.05.

## 3. Results

Altogether 735 out of 994 subjects were identified in the selection process exhibiting good representation of some of the observed respiratory events. The number of analyzed subjects per condition that fulfil the minimum length requirement was: 446 patients with RERA (out of 951 total subjects with this type of event), 536 with HA (out of 950), 281 with OSA (out of 861) and 170 with CA (out of 847).

**Figure 5:**
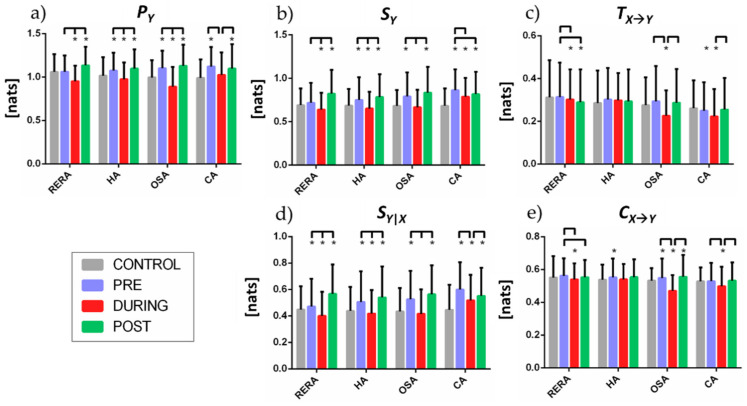
(**a**) Predictive information and its decomposition in terms of (**b**) information storage and (**c**) information transfer, and in terms of (**d**) internal information and (**e**) cross information, for different breathing disorder events and at different phases (CONTROL, PRE, DURING, POST). Statistical significance (*p* < 0.05) between the time segments is indicated by a bridging line, whereas statistical significance (*p* < 0.05) in pairwise comparisons between each segment and CONTROL is indicated by *.

The predictive information of the RR dynamics (Figure 5a) exhibits statistically significant decrease in the DURING period with respect to both PRE and POST for all respiratory events. In HA and OSA the predictive information of the CONTROL segments is lower than PRE and POST, and higher than DURING; these differences are not statistically significant for PRE vs. CONTROL in RERA, and for DURING vs. CONTROL in CA.

The information storage (Figure 5b) displays similar trends to those observed for the predictive information, with values increasing in the PRE and POST segments, and decreasing during the respiratory events, compared to the CONTROL condition. Exceptions to these trends are observed for CA, where the IS higher during the event than during CONTROL and does not change significantly in the POST phase, and in OSA where the information storage does not change significantly between CONTROL and DURING.

The information transfer (Figure 5c) decreases significantly DURING OSA events, compared with PRE, POST and CONTROL, while DURING and POST RERA events it is significantly lower than in PRE or in CONTROL segments. Variations are less evident during CA, and absent during HA.

The direction of changes in internal information dynamics (Figure 5d) is aligned with the changes in IS in all the conditions, with a larger magnitude of the changes. Compared to CONTROL segments, the values of the conditional self-entropy in PRE and POST are always significantly higher, and the values in DURING are lower for RERA and HA, comparable for OSA, and higher for CA. For all types of event, the conditional self-entropy decreases significantly during the event and returns to higher values after the event.

The changes in cross information (Figure 5e) have similar trends to the information transfer, showing a significant decrease during the event (below the CONTROL values) for CA and particularly for OSA; similar changes are observed also for RERA, without the recovery of values in the POST phase, while no substantial variations are observed for HA.

**Figure 6:**
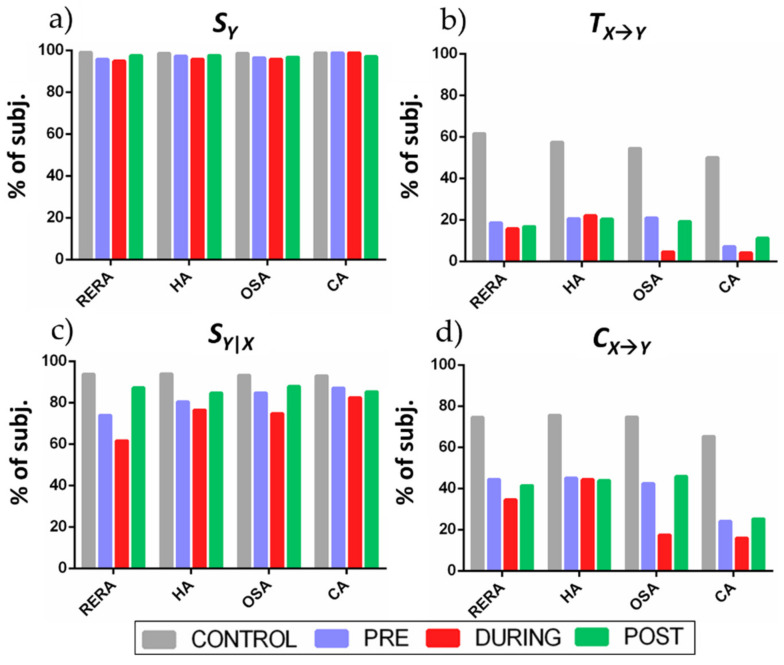
Statistical significance measured using a one-sided surrogate test for: (**a**) information storage, (**b**) information transfer, (**c**) internal information and (**d**) cross information, for different breathing disorder events and at different phases (CONTROL, PRE, DURING, POST).

Figure 6 depicts the percentage of subjects for which the information measures were detected as statistically significant for each condition and phase. A measure for a certain subject is reported as significant if the surrogate test was passed for at least one 300-point sequence forming the overall observation matrix for that subject.

The information storage measure (Figure 6a) was significant in most (>90%) of the subjects regardless of the condition and phase analyzed; the internal information (Figure 6c) displays similar results for POST and CONTROL, with lower percentage in the PRE and DURING segments of each event, especially for RERA episodes. The information transfer (Figure 6b) and the cross information (Figure 6c) show generally low percentages, with cross information being higher, and with trends following those of the information measures shown in Figure 5; in particular, the percentage of subjects with significant cardiorespiratory interaction was almost constant across PRE, DURING and POST conditions for HA episodes, showed a slight decrease during RERA episodes and a more marked drop during OSA episodes, and was the lowest for CA episodes. CONTROL segments showed the greatest scores (>60%), which is expected as a lot more sequences were present and most of those sequences were collected uninterruptedly.

## 4. Discussion

The results described in the previous section highlight the usefulness of exploiting information decomposition to investigate the alterations induced in heart rate and cardiorespiratory dynamics by sleep-related breathing disorders. The predictive information (Figure 5a) is significantly decreased during RERA and all the observed apneic events as compared to the time windows immediately PRE and POST the event, and also if compared to the segments of undisturbed sleep (CONTROL), apart than in case of CA. The periods preceding apneic events (HA, OSA and CA) exhibit higher predictive information as compared to periods of undisturbed sleep, which might serve as a useful marker for detection of these events.

Decomposing the predictive information has allowed to highlight that the cSE values (Figure 5d) present trends similar to self-entropy (Figure 5b), having related meaning, i.e., cSE quantifies regularity of the heart period dynamics, after taking at first the information of respiration into account (see [25] for further details). Both information storage (Figure 5b) and internal information (Figure 5d) are strongly affected regardless of the typology of the sleep-related breathing disorder, with statistically significant decrease during the respiratory effort, indicating a reduced regularity and thus a higher complexity of the RR dynamics. Such findings are reinforced by the statistically significant differences found in almost any phase if compared to CONTROL segments (apart than in DURING segments in OSA for both measures and in PRE segments in RERA with regard to self-entropy) and by the high statistical significance based on surrogate data analysis (Figure 6c). These results are in agreement with previous findings [16] reporting that severe apnea–hypopnea increases the complexity of the nocturnal dynamics of the cardiovascular system, significantly reducing the predictability of the time courses of the parasympathetic component of RR. Physiologically, the reduced internal information during the event is indicative of an increased predictability of the target by its past above and beyond the part that was predicted by the past of the driver, which can be ascribed to a reduced regularity of the heart period unrelated to its coupling with respiration. The decrease of both information storage and internal information in the DURING phase is followed by an increased value in the POST phase, evidencing a compensation that occurs just after the event to recover to values close to the PRE condition. These findings highlight that the loss of predictable information occurring during the breathing disorders is a transient phenomenon, which reflects the alterations in the respiratory patterns caused by the respiratory effort. On the other hand, both the self entropy and the conditional self entropy measured during all phases of the respiratory events (PRE, DURING, POST) showed significantly higher values when compared to the CONTROL segments. This result documents a generally higher predictability of the cardiac dynamics associated with breathing-related sleep disorders, which can be associated with an activation of the sympathetic nervous system during the episodes [22,40,50].

The decomposition of the predictive information evidenced also measures of the information shared between the heart period and the respiratory dynamics, which physiologically reflect cardiorespiratory interactions and the so-called respiratory sinus arrhythmia, RSA [51,52]). CE values (Figure 5e) present trends similar to TE (Figure 5c), since C_X->Y_ quantifies the part of the information carried by the present of RR that can be predicted exclusively by the past of RESP, and this behavior is also expected given the presumed unidirectional nature of cardiorespiratory interactions [24,53]. The CE has been already demonstrated to represent a proper measure of the overall directed interactions from driver to target when their nature is unidirectional [24], and thus can be regarded as a simpler and more effective approach in this context, returning more significant values (compare Figure 6d vs. Figure 6b).

In detail, analyzing the patterns of information transfer (Figure 5c) and cross-information (Figure 5e) it is possible to infer that cardiorespiratory interactions are altered differently according to the specific breathing disorder: apneic events tend to reduce such interactions, which are then restored in the POST phase. Statistically significant decreases of both measures are reported in the DURING phases in case of RERA, CA and OSA events. This may be due to the increased sympathetic modulation during episodes of apnea, which provoke heart rate variations not modulated by the respiration, i.e., reduced RSA during sleep-related breathing disorders [54,55]. Such results are in accordance with previous findings in the literature which reported reduced cardiorespiratory interactions during OSA, assessed in terms of information transfer [23] or other techniques such as orthogonal subspace projections [5], cardio-respiratory phase-locking [20] or cross-correlation between frequency components of respiration and HRV [56]. OSA presents the most marked and durable variations in terms of information transfer, with statistically significant differences not only during the events, but also before and after, when compared to CONTROL; as this apneic disorder represents a risk factor for cardiovascular illnesses [57], the marked changes in cardiorespiratory interactions detected by TE and especially CE could be exploited as a marker of such risk. On the other hand, in milder obstructive events such as HA, cardiorespiratory interactions are preserved, since no statistically significant different variations of either information transfer or cross information have been detected in DURING phase if compared to CONTROL segments. This result may appear in disagreement with that found in [23] where a reduced information transfer was found even during milder obstructive events, but such a finding may be due to the use of ECG-derived respiratory signals (EDR), since the small variations that might be present in the respiratory effort during hypopneas are not captured by the EDR signal. Conversely, in [5] the hypopneas appeared similar to normal activity in terms of heart rate modulation power, thus inferring sustained cardiorespiratory interactions. Such results demonstrate that the most difficult apneic respiratory events to classify are hypopneas, since they may appear similar to normal segments being not always accompanied by arousal, desaturation, snoring, or thoraco–abdominal paradox [5].

It is physiologically important to evidence that the information transfer and cross information after RERAs are not fully recovered to PRE values, thus documenting the preservation of depressed cardiorespiratory interactions, contrary to what occurs after an OSA or CA event. Just after a RERA event, the information transfer is also statistically different if compared to CONTROL segments, and this behavior is not observed (with regard to T_X->Y_) in any other respiratory sleep disorder. Such results may add clinical relevance to RERA events, which has been debated in the literature [58], since the effects of RERA episodes are surely not evident as OSA (being not associated to concomitant oxygen desaturation), but are longer-lasting in terms of variation of cardiorespiratory interactions. This suggests that RERA events may be indicative of the development of more serious apneic events in a later stage if unnoticed and inadequately addressed [29,30] and may reinforce the still not definitely confirmed findings which relate RERA events with the development of cardiovascular comorbidities, which have already been suggested in [59] reporting repetitive increases in blood pressure concomitant with RERA episodes during sleep.

Even if our study was not focused on an automatic classification task of different sleep-related breathing disorders, the obtained results appear promising for employing the proposed measures as discriminative features. Up to now, the recommendations and rules for scoring of different apneic events and RERAs include airflow signal, blood oxygen saturation, thoraco-abdominal belts and EEG for sleep monitoring in polysomnography recordings [9]. Recently, some studies suggested that HRV parameters are powerful tools for screening of OSAs events avoiding the need for polysomnography monitoring [60,61]. However, to our best knowledge, no indices based only on ECG or assessment of cardiorespiratory interactions have been proposed so far with a discriminative potential to identify all types of apneic events and RERAs.

A methodological challenge faced in this study is the model-free estimation of information dynamic measures from the short-length data available during the observed instances of sleep breathing disorders. To obtain reliable estimates of information dynamics, we combined the development of fuzzy kernel estimates, the use of low-dimensional embedding variables, and the implementation of an approach to collect observations of these variables from repeated instances of the same experimental condition. Nevertheless, the measures of cardiorespiratory interaction (the TE in particular) showed a generally low statistical significance (Figure 6). This behavior is likely mostly due to the fact that observations are collected from different segments which are assumed to be repetitions of the same stationary dynamics, which may not be always true. On the other hand, this way to proceed is the only feasible in order to collect enough realizations of the observed variables under stationary conditions; non-stationary approaches employing time-varying techniques are also applicable, but they are mostly restricted to linear analysis and typically provide less consistent measures because such measures are derived from few data points [62,63]. This issue is not present for CONTROL segments which are collected from several contiguous segments, thus showing more significant values in our results. Other possible limitations of the study are represented by the dataset and the non-controlled environment in which the signals have been acquired. In detail, the used dataset has been difficult to be analyzed due to the inhomogeneous distribution of the breathing effort events and the high variability shown across the subjects, while we assume that the underlying information flow behaves similarly across all patients. Conversely, a controlled environment for the signal acquisition procedure (e.g., taking care that the subject never moves during CONTROL phases) would probably lead to more reliable or conclusive results.

Future studies may envisage the extension of the proposed analysis taking into account more variables recorded through polysomnography (e.g., electroencephalogram, electromyogram or electrooculogram signals) to account for additional regulatory mechanisms and exploiting in full the network physiology approach [18,64]. This may provide further information on how breathing disorders also affect other systems (e.g., cerebral) [65,66] by estimating changes in their mutual interactions. Moreover, a future work may focus on training a classification model to identify and classify different apneic events and RERAs in polysomnographic recordings, incorporating the measures evaluated in this study that exhibit statistically significant differences if compared to the control segments in addition to standard features derived from heart rate and respiration time series. A simulation study on cardiovascular interactions on sleep data using the fuzzy kernel estimator may be also envisaged. Finally, a multiscale information decomposition to quantify the amount of information transferred from blood pressure and respiration (sources) to the RR (target) [41] may supplement further useful information on cardiovascular autonomic control during different breathing disorders.

## 5. Conclusions

The aim of this paper was to conduct a cardiorespiratory analysis during breath-related sleep disorders via information dynamics. Our results document that respiration-related breathing disorders during sleep cause noteworthy changes in cardiorespiratory information dynamics and interactions which can be successfully described using information-theoretic measures. Such variations depend on the type of disorder and are particularly evident during the event compared to the baseline condition, but also persist for several seconds after the breathing disorder. These findings can have clinical implications, resulting in perspective useful for the differential classification of respiration-related breathing disorders.

Physiologically, the results confirm that sleep disorders are associated with impaired cardiovascular autonomic control, thus provoking an increased probability of developing cardiovascular diseases, also representing a risk factor for the insurgence of other chronic diseases or even more life-threatening events.

## Data Availability

Publicly available datasets were analyzed in this study, i.e., the open-source database provided for the Physionet/CinC Challenge 2018. This data can be found here: https://archive.physionet.org/physiobank/database/challenge/2018/ (accessed on 20 May 2021). The codes developed for the analyses carried out in this work are available upon request.
